# Exploring isolation, self-directed care and extensive follow-up: factors heightening the health and safety risks of bariatric surgery abroad among Canadian medical tourists

**DOI:** 10.1080/17482631.2019.1613874

**Published:** 2019-05-14

**Authors:** Carly Jackson, Jeremy Snyder, Valorie A. Crooks, M. Ruth Lavergne

**Affiliations:** a Faculty of Health Sciences, Simon Fraser University, Burnaby, British Columbia, Canada; b Department of Geography, Simon Fraser University, Burnaby, British Columbia, Canada

**Keywords:** Medical tourism, Canada, bariatric surgery, risk, challenges

## Abstract

**Purpose:** This article explores first-hand accounts of Canadian bariatric patients’ experiences of seeking and obtaining weight loss surgery abroad through the practice of medical tourism. While researchers have identified many of the challenges and associated health and safety risks imposed on patients by engaging in medical tourism generally, little is known about the specific challenges experienced by Canadians seeking bariatric surgery abroad.**Method:** To better understand these challenges, we conducted thematic analysis on interviews conducted with 20 former Canadian bariatric tourists.**Results:** Our analysis illuminated three key challenges Canadians face in obtaining bariatric care: (1) stigma and isolation from friends, family and medical professionals; (2) self-directed navigation of domestic and destination health care systems; and (3) challenges with obtaining adequate follow-up care in Canada.**Conclusions:** While these challenges identified by participants may occur in other forms of medical tourism, it appears that these challenges are occurring simultaneously in cases of bariatric tourism by Canadians. These challenges appear to work in conjunction to heighten the health and safety risks potential Canadian bariatric tourists may be exposed to. Unless structural changes occur to increase domestic availability of bariatric surgery, Canadians are likely to continue seeking this care abroad.

## Introduction

In the 21^st^ century, obesity and weight-related morbidities have become some of the leading causes of chronic disease worldwide. According to the World Health Organization, globally in 2016 more than 1.9 billion adults were classified as overweight while 650 million were classified as obese^1^ (World Health Organization, 2018). The number of overweight and obese adults in Canada reflect these global statistics. Canadian statistics in 2017 indicate that 9.6 million adults were identified as overweight while an additional 7.2 million adults were classified as obese, representing 26% and 19% of the Canadian population respectively (Statistics Canada, 2018).

Excess weight gain can lead to many serious obesity-related negative health outcomes, which can contribute to a reduced quality of life as well as a reduced life expectancy if left untreated (Buchwald, Ikramuddin, Dorman, Schone, & Dixon, 2011). Negative health outcomes can include: weight-related chronic conditions, such as type II diabetes or atherosclerosis (Buchwald et al., 2011; Nguyen et al., 2011; Runkel et al., 2011); mental health disorders, such as depression or anxiety (Green, Engel, & Mitchell, 2014); and social discrimination due to stigmatization (Melin, Reynisdottir, Berglund, Zamfir, & Karlström, 2006).

Given the multifaceted negative health outcomes that are associated with being above a healthy weight, weight management strategies are being increasingly examined in the public health, medical, and health services literatures. For example, it has been consistently shown that for both overweight and obese individuals, conventional treatments of diet and exercise are generally not effective for eliciting weight loss over the long term (Karlsson, Taft, Rydén, Sjöström, & Sullivan, 2007; Moroshko, Brennan, & O’Brien, 2012; Runkel et al., 2011). When these conventional treatments fail, some individuals look to surgical options. Weight loss surgery, also known as bariatric surgery, refers to a collection of surgeries designed to induce weight loss through systematic changes to the digestive system. (Buchwald, Estok, Fahrbach, Banel, & Sledge, 2007).

There are many factors that act as barriers and, therefore, limit access to bariatric surgery in Canada. First, following recommendations from the 1991 National Institute of Health consensus conference, Canada requires a person to have a BMI greater than 40 kg/m^2^ or a BMI between 35 and 39.9kg/m^2^ if they have at least one significant weight-related co-morbidity to qualify for surgery (Christou & Efthimiou, 2009; National Institute for Health, 1991). Second, provincial variation in funding and resources dedicated towards bariatric surgeries constrain access to care to varying degrees across the country. Finally, prospective patients must also provide proof of a history of failed attempts at weight loss through conventional diet and exercise regimes, as well undergo an evaluation process with a nutritionist/dietician, a psychologist and a bariatric specialist (Christou & Efthimiou, 2009). This extensive pre-operative process, coupled with resource constraints, leads to a national average wait time of approximately five years from time of initial office visit with the bariatric specialist to the surgical date (Canadian Institute for Health Information (CIHR), 2014; Christou & Efthimiou, 2009).

Challenges related to eligibility criteria and timely access to care, among other factors, have prompted some Canadians to seek bariatric surgery outside of the country through the practice of medical tourism (Birch, Vu, Karmali, Stoklossa, & Sharma, 2010; Kim, Sheppard, de Gara, Karmali, & Birch, 2015; Sheppard et al., 2014a; Sheppard, Lester, Karmali, de Gara, & Birch, 2014b). This care is paid for out of pocket and is beyond any government administered cross-border care arrangements (Adams, Snyder, Crooks, & Johnston, 2013). While it is currently unclear exactly how many Canadians are engaging in medical tourism, media coverage and internet discussion has indicated that many bariatric patients unable to receive care in Canada are indeed travelling abroad for such care (Goh, 2016; Kirkey, 2016; Pagel & Berry, 2016). Media and academic discourse have highlighted that Mexico is, by far, the most popular destination for bariatric tourism by North Americans, given lower costs and close proximity for travel (Goh, 2016; Kim et al., 2015; Kirkey, 2016; Pagel & Berry, 2016). Another popular destination country for Canadians seeking bariatric surgery abroad is the USA, although surgeries are, on average, more expensive than in Mexico and consequently, less Canadians pursue surgery here compared to Mexico.

It should be noted that limited private options for bariatric care are available in Canada. The adjustable gastric band, for example, is readily available in private clinics across Canada, often with shorter accompanying wait times than typically seen in the Canadian public system. This can largely be attributed to fewer required pre-operative visits with specialists in private clinics (Martin, Klemensberg, Klein, Urbach, & Bell, 2011). However, the lap band in a private clinic in Canada averages $16,000, compared to no direct cost through the public system (Martin et al., 2011). Therefore, while private domestic options offer shorter wait times than typically experienced in the public system, the higher out-of-pocket payments than what is required in some medical tourism destinations appears to further drive patients to seek this care abroad. Additionally, patients that obtain bariatric surgery privately in Canada are not entitled to the post-operative programme offered through the public system. These patients would be limited to any post-operative care that is offered by the private clinic, which is the same situation they would experience by obtaining the surgery internationally.

Much of the existing research about bariatric surgery pursued by Canadian medical tourists has focused on the potential financial burden on the public health care system when they present complications upon return to Canada, which poses financial risks to this system (Birch et al., 2010; Kim et al., 2015; Sheppard et al., 2014a, 2014b). This research has shown that such complications can become quite costly for these patients’ local health systems even though they individually chose to exit these systems to privately purchase surgery in another country.

Studies examining the practice of medical tourism have found some health and safety risks for patients that are either distinctive or amplified when compared to the context of accessing surgery in their home countries (Crooks, Kingsbury, Snyder, & Johnston, 2010; Johnston, Crooks, Snyder, & Dharamsi, 2013; Lunt & Mannion, 2014; Turner, 2010). In the context of this analysis, a health risk is any risk to the patients’ physical or mental health that may be amplified or introduced through the act of medical tourism. For example, flying post-operatively or with a serious medical condition can put a patient at risk for deep vein thrombosis, a serious medical condition that can lead to pulmonary embolism (Lunt & Mannion, 2014). Medical tourists may also be at an increased risk of contracting an infection in a facility abroad or straining their recovery by engaging in tourist activities post-surgery or during their travels home (Crooks et al., 2010). Continuity of care is another concern for medical tourists as patient medical records are not often exchanged between the home and the destination physicians, consequently undermining continuity of care (Turner, 2007). Should complications arise upon return home, communication with the facility abroad may be challenging due to potential language barriers, differing clinical or surgical standards of practice and/or challenges with time zones and operating hours (Turner, 2010). In the context of this analysis, a safety risk encompasses any element of the travel aspect of medical tourism that may increase the risk to a patient’s safety. For example, a patient’s safety may be at risk through travelling to an unfamiliar country.

This existing research on the health and safety risks associated with medical tourism has not explored the specific context of bariatric surgery. We believe this is a pressing knowledge gap given the growing global popularity of these surgeries, the increasing number of clinics worldwide seeking to attract international patients, and the fact that, when performed domestically, such interventions are typically coupled with therapy and nutritional counselling as well as post-operative follow-up that cannot typically be incorporated into time-limited trips abroad for surgery (Birch et al., 2010; Glenn, McGannon, & Spence, 2012; Salant & Santry, 2006; Snyder & Crooks, 2010). Broader research regarding Canadians’ involvement in medical tourism has shown that physicians can be reluctant to treat patients post-operatively upon their return home due to lack of familiarity with the procedure obtained abroad, concerns about liability, and challenges regarding informational continuity of care, among other factors (Johnston et al., 2013). Bariatric surgery already runs an elevated risk of surgical and post-operative complications, even when it is performed domestically. The risk of these complications occurring may be heightened when the surgery is performed out of the country (Birch et al., 2010). Given this, medical professionals in Canada may be especially reluctant to assist with follow-up care in cases of bariatric tourism, although it is currently unknown to what extent this is occurring in Canada.

The current analysis takes a departure from the existing research focus on surgical costs and complications among Canadians who have undertaken bariatric surgery abroad through examining first-hand accounts that provide new experiential insight into this transnational care practice. We do so by thematically examining 20 qualitative interviews conducted with Canadians who had previously travelled to Mexico for bariatric surgery to identify specific health and safety risk factors anticipated, encountered, or avoided throughout this journey.

## Methods

### Recruitment

After obtaining ethics approval from Simon Fraser University, we sought to recruit Canadian residents who had previously travelled, within the last 10 years, outside of Canada to obtain bariatric surgical procedures. Canada presently has no system for tracking individuals who leave the country each year for medical procedures and, therefore, social media was used as the primary medium through which recruitment was conducted. Prospective participants were informed of the study through targeted advertisements placed on social media websites, namely Facebook and other online patient support forums. In addition, an article about the study was published on a popular Canadian obesity blog. Finally, participants were asked to provide study details to others in their networks that may qualify for the study and be interested in participating.

### Data collection

Interviews with former Canadian bariatric tourists were conducted by phone between February and May of 2016. Each interview ran for approximately 30–50 min. These interviews used a semi-structured interview guide, thereby allowing participants to elaborate on questions they find especially salient or pertinent to their experience. The interview script was developed through a review of literature related to medical tourism, bariatric surgical standards and practice, and bariatric tourism. Questions in the interviews were designed to capture the planning, travel and after-care experience. In addition, reactions/support from family, friends, and members of the Canadian medical system were probed. All the interviews were conducted by the lead author to enhance consistency (McGrath, Palmgren, & Liljedahl, 2018). To minimize the impact of researcher bias on the generation of the data, the semi-structured interview script was used after being developed and approved by the research team. Further, having the same author conduct all the interviews allowed for similar follow-up questions to be pursued over the course of the interviews.

### Analysis

All interviews were digitally recorded and then transcribed verbatim. Using Braun and Clarke’s conceptualization, thematic analysis was undertaken to identify, analyse and subsequently, report key themes in the data (Braun & Clarke, 2006). The first step in analysis was independent review of the transcripts by the three members of the team. Through this review the authors reviewing the transcripts independently identified emerging themes, concepts and issues brought forth by participants, thereby minimizing the potential for researcher bias in interpretation the data. In the second step, the outcomes of the independent review were then examined collectively, and the themes, concepts, and issues were compared to identify areas of overlap (Braun & Clarke, 2006). Then a codebook of initial codes was generated from the areas of overlap identified by the team. The lead author then re-analysed all the transcripts and coded each in relation to the codebook, adding to and refining the codes as needed. In the third step, patterns in the codes were identified and then grouped into overarching themes. Three main meta-themes were identified that form the basis of this analysis. The fourth and fifth steps in our analytic process, encompassed reviewing and refining these overarching themes to ensure no overlap between the themes (Braun & Clarke, 2006). In the final step, the lead author then re-examined all the transcripts to identify excerpts relevant to each theme, which were later reviewed by the other authors to confirm the scope of each theme.

## Results

A total of 20 interviews were conducted with individuals who had obtained bariatric surgery outside of Canada. All participants were Canadian residents who qualified for provincially funded health care. Participants were residing in British Columbia (n = 2), Alberta (n = 4), Saskatchewan (n = 12), Manitoba (n = 1) and Nova Scotia (n = 1) at the time of seeking surgery. Most participants travelled for the vertical sleeve gastrectomy procedure (n = 16). Other procedures included: adjustable gastric band (n = 1), gastric plication (n = 2) and Roux-en-Y (RNY) gastric bypass (n = 1). All participants obtained their respective surgeries in Mexico. The average length of time between participants respective surgeries and interview was 3.9 years, with a minimum time between of two months and a maximum time of 8 years.

Through the participant interviews, three meta-themes emerged that appear to indicate that Canadians who engage in bariatric tourism can experience heightened health and safety risks when compared to having the surgery performed domestically. The first of these themes is feelings of isolation and stigma from family and friends, as well as health care professionals. These feelings led to many of the participants having to engage in self-navigation of care, the second theme identified in our analysis. Participants were found to have self-navigated not only the potential bariatric services offered by the Canadian health care system, but also the private system in Mexico. A final theme is the extensive, life-long follow-up care necessary for bariatric procedures, which, when coupled with isolation and self-navigation, led many participants to receive inadequate follow-up care. In the remainder of this section we expand on these thematic findings. See Table I for an overview of the challenges discussed in the remainder of this section and their associated health and safety risks.

**Table I.** Relationship between bariatric tourism challenges and associated health and safety risks.

	Challenge	Challenge in Context	Associated Health and Safety Risks
1	Stigma and Isolation	Experiences of stigma and isolation from family, friends, and medical professionals in the Canadian health care system	i. *Risk of (dis)continuity of care* ii. *Risk of lack of informed consent*
2	Lack of familiarity with Mexico	Participants’ pre-conceived understandings of the safety of Mexico and standard surgical procedures	i. *Risk of lack of informed consent* (language barriers)
3	Self-navigation of care	Surgical options and care obtainment identified by participant in both home and destination countries	i. *Risk of medical complications* ii. *Risk of (dis)continuity of care*
4	Information seeking primarily online	Data on procedures, clinics and health care professionals abroad obtained through online mediums	i. *Risk of lack of informed consent* *ii. Risk of poor outcomes*
5	Reliance on anecdotal information	Former patients’ testimonials on surgical outcomes and experiences of care abroad heavily relied upon by patients	i. *Risk of lack of informed consent* *ii. Risk of poor outcomes*
6	Inadequate follow-up care	The multi-disciplinary care required for strongest health outcomes in bariatric surgery is largely missing due to the international dynamic or lack of support from home physician	i. *Risk of (dis)continuity of care* ii. *Risk of medical complications*

### Stigmatization and isolation from family, friends and medical professionals

The first challenge leading to heightened health and safety risk potential are feelings of isolation and stigma, which led some participants to not tell others in their immediate support networks of their decision to go abroad for surgery. This silence around the surgery continued long after returning to Canada, with many participants echoing similar sentiments of “*I have told very few people that I had, that I had weight loss surgery.”* Or, as one participant stated: “*My family still don’t know.”* In many cases, participants never told friends or family. Nine participants spoke directly to the stigma associated with being overweight or obese as the primary reason for not informing many friends or family about their decision to undergo surgery:

*I’ve been very private about the fact that I had to have this surgery. So I’ve been private intentionally because I don’t feel that it’s anyone else’s business. I think there’s a lot of judgement around obesity and overweight and interventions as such. And I don’t particularly feel like addressing those*.

Another participant detailed the very negative reaction she received from telling a family member, “*…he’s a paramedic and he was just furious when he heard, yeah just furious, because of the stigma behind it.”* Fear of negative or stigmatizing reactions from family and friends worked to further enforce the isolation these individuals already feel in relation to their weight and weight-related health conditions.

Nine participants discussed feelings of shame and stigma directed from domestic health care professionals that worked to dismiss bariatric surgery as a viable option to obtain domestically. Variations of the mantra *“eat less, exercise more, watch your portions”* were generally the medical advice given to participants from Canadian health professionals when they first started exploring surgical options, even after years of failed diet attempts. One participant explained:

weight would be assessed annually, it would be noted you need to lose weight or there was treatment of hypertension but there was really never any particular support or discussion around achieving it beyond sort of the usual, move more eat less… There was actual dismissal of bariatric surgery as being a viable option by my family physician.

This participant felt very isolated from the Canadian health care system as it was not seen as addressing her health care needs despite repeated requests and failed attempts to lose weight through conventional weight loss programmes. Although her weight–related health conditions, such as hypertension, would be treated, she still felt that her health care professionals seemed dismissive of her broader weight issues. While these feelings of isolation and stigma from the Canadian health care system may have been experienced even if the participant had been able to move forward with receiving bariatric care in Canada, there is a sense that obtaining care abroad heightens the stigma and isolation that is attached to the surgery. As one participant explained, there is *“less of a stigma attached to having the surgery done here [Canada].”*

Perceptions about the dangers of travelling to Mexico for surgery led to 11 participants telling few, if any, of their friends, family, or medical professionals about their intention to obtain, let alone travel to Mexico for, the surgery. For participants that did tell family or friends of their intentions, reactions were quite often negative towards Mexico as a surgical destination. For example, one participant discussed her experience of trying to tell a friend she was going to Mexico for the surgery:

I have one friend of mine… she did not like the idea of me at all going to Mexico and having it done and she understood that I wanted to be healthier and have a better life for myself but the whole idea of me going to Mexico definitely scared her.

Other participants that chose to share their decision to travel with their support networks felt they had to wait until they were in Mexico before they could tell anyone about their decisions to go abroad for surgery. As one participant noted “*I told one sister when I was actually in Mexico and that’s it.”* For these participants, perceived stigma from loved ones regarding the lack of safety of Mexico led to a lack of support from family and friends over the decision to travel for the surgery.

### Self-navigation of domestic and destination health care systems

Ten participants discussed experiences of having to self-navigate their own bariatric care, first domestically and then again with the care they received in Mexico. In the context of this analysis, self-navigation is being taken to encompass all aspects of care obtainment that participants were left to do on their own, either with limited or no support and guidance from a medical professional. As discussed in the previous sub-section, many participants noted feeling unable to talk to their regular health care professionals due to negative perceptions surrounding bariatric surgery as a viable option. One participant discussed the negative reaction she received from her family physician regarding pursuing bariatric surgery, as the family physician only agreed to remain her doctor if she would

go to this other private weight management clinic and be followed by them, because he said we all know now that if you have that surgery you are going to be a burden on the health care system now for the rest of your life and you will have to be closely monitored.

Similarly, another participant stated, *“There wasn’t any assistance from the GP’s [family doctor] office, in fact he kind of frowned upon my suggestion of seeking bariatric care.”* For these participants, their regular health care providers were not supportive of their desire to seek a surgical solution to their weight related health problems, leaving them to find self-directed ways of accessing the surgical care.

In many cases, due to the lack of regular physician support or knowledge of the field, participants noted having to do most or all of the information seeking about bariatric surgeries on their own:

it was really doing my own homework and figuring out for myself that I needed to act now before the co-morbidities and other health issues were going to arise and that you know, just sort of broaden my own understanding… And so, yeah, I did my own work around that.

Thus, once participants understood they were not going to be able to receive their desired bariatric surgery through the Canadian health care system, many were left having to self-navigate and rely on themselves to make up for the lack of information provided by professionals within the Canadian health system.

The primary outlet the vast majority of participants (17 of 20) turned to for information was the internet. Many noted seeking advice from previous bariatric tourists from Canada and the USA through the use of social networking sites, namely through bariatric surgery information and support groups on Facebook and through blogs/forums. As one participant explained:

So I based my end decision… on the success of other patrons and their feedback on the procedure itself, and the locations, the surgeons. I joined a weight loss forum online, a chat group and people comparing notes and people were brutally honest about which surgeons they liked and who they didn’t like eventually.

Another explained: *“I used what this woman had told me [online]. She told me the clinic she went to, the doctor, and then I went and researched from there. But I totally relied on this woman.”* Notably, information seeking online did allow some participants to learn that local surgeons practicing bariatric surgery did not have the experience level they desired, thus further entrenching their plans to seek surgery outside of Canada. As one participant explained her extensive information seeking processes allowed her to *“[get] a bit of understanding of what is available around the zone that I lived…then I began to look outside of my province and outside of the country for that.”* Lack of available local expertise seemed to further entrench the belief that Canada was not a viable option for their desired bariatric surgery and pushed patients to self-navigate for where they could find the level of expertise they desired.

### Inadequate follow-up care domestically

In Canada, the public health care system requires an extensive pre-operative and subsequent post-operative programme for all bariatric patients. Participants bypassed these required steps by travelling abroad for the surgery. For example, as one participant explained:

I’ve read several things about the process in Canada and you know they give you a diet and see whether you can follow that procedure for a year and then there’s some emotional, some counselling for another couple of years, and then, you know, wait another year and see whether you still want to do it or not. There’s lot of pre-scanning that’s done before you even get a chance to get in and do your surgery and you would probably be part of a group when you came out. Whereas having had it done in Mexico, I didn’t.

Another participant discussed the implications of bypassing these steps, explaining that if she had been

able to get [the surgery] in Canada, the pluses would have been: have more professional staff available, more educated staff available. Like dieticians and people that were more familiar in the care, the more immediate care…. More psychiatry. All those kinds of things more available right away if you needed it as opposed to in Mexico you don’t really have that.

While these participants desired to bypass the extensive pre-operative programme in Canada and thereby get their procedures done more quickly, doing so meant that they were not able to access the standard post-operative care in Canada. In some instances this included not receiving information about local in-person support groups for those recovering from bariatric surgery, which some participants would like to have attended.

In some cases where participants’ regular physicians, and specifically family doctors, supported their decisions to go to Mexico for bariatric surgery, accessing aftercare services domestically became possible. For example, one participant detailed her extensive pre-operative discussion she had with her physician in Canada and the “game plan” they had in place should complications arise:

My primary care physician and I walked through what was, what would happen, in theory, if I had complications and it was one of the known risks that I entered into it with, in facing the surgery. If I had the surgery, had a complication and then had to come back here, and so my primary care physician and I discussed that I would have to be referred through emergency to a local surgeon. They would have to know what my surgery was and so on and so forth.

Another participant had a very different experience:

My GP wasn’t particularly interested in providing any follow up care. He said, “you saw a surgeon in another country and you should be following up with them.” So, but I didn’t have any problems thankfully, ‘cause that could have been a serious issue if the GP wasn’t cooperating and I’m having medical issues, right?

Information seeking online and self-navigation of health care affected not only decisions regarding where to travel and which surgery to obtain, but also impacted the types of follow-up care information participants received and acted on. As one participant explained, he felt he was: *“pretty informed when I walked in the door, just by looking at all these other websites.”* Similar sentiments were echoed by other participants. This belief that the internet was sufficient to inform their medical decision making, both pre-operatively and post-operatively, led many participants to not seek any formal follow-up care upon their return to Canada.

## Discussion

The themes identified in this analysis provide a strong indication that people who obtain bariatric surgery outside of Canada face many challenges. We contend that these challenges can introduce or exacerbate health and safety risks—again refer to Table I for an overview of the relationship between the challenges discussed in this analysis and their associated health and safety risks. For the participants, stigma regarding their above average weight, coupled with the notion that bariatric surgery is the “easy way out,” led many to feel isolated from their family, friends, and the Canadian health care system in general. For many participants, experiencing stigma and isolation from medical professionals led to harms in the doctor–patient relationship, meaning patients were unable to or faced challenges in confirming medical information with their family physicians, thereby risking harm to informed consent and significant disruptions in continuity of care. Several participants discussed facing this challenge in confirming this medical information with their family physicians both before travelling for surgery and after.

Confirming previous research, Mexico appears to be a popular destination for bariatric tourists (Kim et al., 2015). As mentioned earlier, Mexico is the most popular destination for bariatric tourism by Canadians due to the lower costs of surgical care, close proximity to Canada leading to low travel costs, and little to no wait times for desired surgeries (Kim et al., 2015). Lack of familiarity with Mexico and/or perceptions of the country appeared to add a new form of stigma and isolation for them to contend with, especially regarding medical professionals that were very uncomfortable with the idea of their patients receiving care in Mexico due to their lack of familiarity with Mexico and their standards for surgical practice.

Feelings of dismissal by the Canadian health care system, coupled with isolation from friends, family, and medical practitioners regarding medical decision-making, left many of these former bariatric patients having to self-navigate their own bariatric health care. For these participants, the health and safety risks associated with self-navigation of care (such as the risk of discontinuity of care or the increased risk for medical complications) were heighted as they were left to self-navigate both the Canadian health care system and their desired care in Mexico. These health and safety risks appeared to stem from a heavy reliance on anecdotal experiences of previous patients or other information found on the internet, and patients faced challenges confirming the validity of this information with their family physicians.

Finally, challenges with accessing follow-up care appeared to be mitigated through support of physicians in Canada or heightened when that support was lacking. When the support of a family physician was lacking, challenges with obtaining adequate follow-up care appeared to heighten the risk to continuity of care and the potential risk of medical complications. Life-long follow-up care is imperative for success with bariatric tourism as medical complications or nutritional deficiencies can occur in a patient any time following surgery (Aills, Blankenship, Buffington, Furtado, & Parrot, 2008; Carter, 2015; Green et al., 2014; Guerreiro & Ribeiro, 2015; Wilson & Datta, 2014). The risk of (dis)continuity of care and/or medical complications did appear to be heightened through the practice of bariatric tourism as many participants discussed experiencing significant challenges associated with family physicians’ unwillingness to refer them for post-operative blood work or scans to ensure proper healing. These challenges appeared to stem from unavailability of resources because of not going through the regimented pre-operative bariatric surgery requirements at home or the lack of support from the family physician over the patient’s decision to obtain this surgery abroad.

Most of the issues central to the three themes identified in this analysis are not, on their own, unique to bariatric tourism or medical tourism more generally. The three key challenges that lead to health and safety risks in the context of bariatric surgery via medical tourism by Canadians are likely to be present when accessing care domestically and/or in other cases of medical tourism. In particular, patients may receive varying degrees of aftercare support, even when the surgery is received domestically (Jumbe & Meyrick, 2018; Meana & Ricciardi, 2008). In other cases of medical tourism, having to self-navigate various health care systems is a common risk that has been associated with potential disruptions in continuity of care or even un/mis-informed decision-making, which threatens patients’ ability to give informed consent. For example, research has shown that individuals seeking plastic surgery or in-vitro fertilization abroad may expose themselves to these same risks (Glenn et al., 2012; Murphy, 2009). Concerns regarding access to domestic aftercare, and therefore risks to continuity of care, for returning medical tourists have also been identified by the broader medical tourism literature (Johnston et al., 2013; Snyder & Crooks, 2010; Turner, 2010), so this too is not unique to bariatric tourism. Existing research suggests that reasons Canadian physicians may be reluctant to coordinate after care for returning medical tourists include their lack of familiarity with the procedure or quality/standards of care abroad, unwillingness to disrupt existing waitlists for such care that are populated by those who had surgery domestically, as well as concerns regarding their own legal liability should complications emerge or become exacerbated (Collier, 2014; Crooks et al., 2013; Runnels et al., 2014; Snyder, Crooks, Johnston, & Dharamsi, 2013).

**Figure 1.**
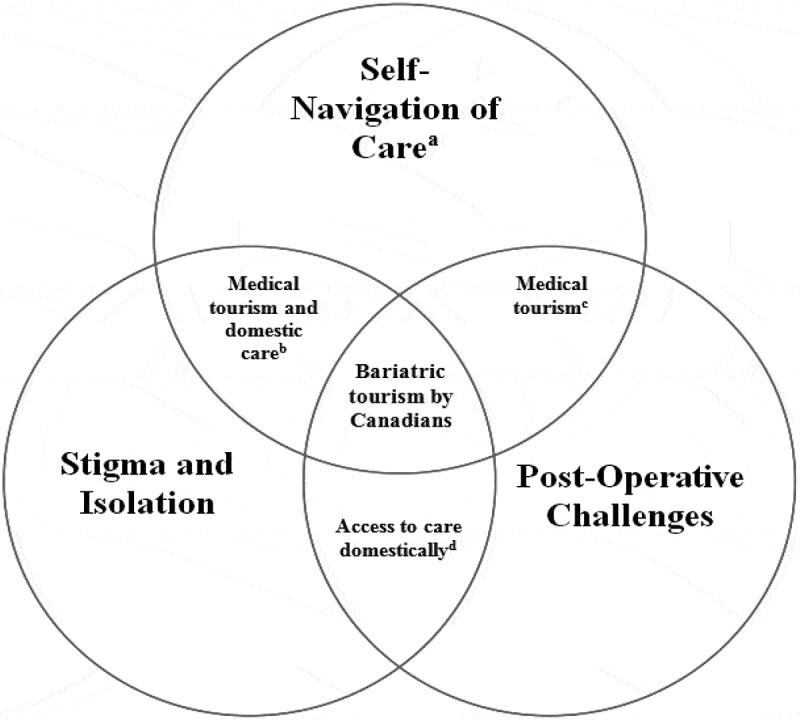
Risk relationships experienced by Canadian bariatric tourists in regard to domestic risks and those heightened by medical tourism. ^a^Both of the domestic and/or destination health care systems.^b^Overlap of risks that are present both when the patient seeks care domestically and through medical tourism.^c^Overlap of risks unique to the context of medical tourism.^d^Overlap of risks primarily experienced when the patient receives care domestically.

While health and safety risks are present with all surgeries, including bariatric surgeries, here we contend that the added dimension of privately pursuing bariatric surgery abroad heightens or amplifies some of these existing risks, while in some cases introducing new ones through navigating the three challenges reported on in the findings. What is clear from this analysis is that bariatric tourists are experiencing challenges *beyond* those that are commonly present when the care is sought domestically in Canada. Not only that, but also most participants experienced some degree of each of the challenges. Therefore, what is at the very least worrisome and also likely distinctive about bariatric tourism by Canadians is that all three of the meta-themes identified in this analysis are working in conjunction, as shown in Figure 1, to potentially heighten patients’ exposure to health and safety risks. These risks are also likely beyond what the patient may experience when the surgery is obtained domestically. We contend that it is this “triple threat” of risks that is faced by Canadian bariatric tourists that makes this practice of going abroad for surgery especially problematic, which we explore in greater detail in the following sub-section.

### Intersecting and overlapping challenges

As this “triple threat” of risks faced by bariatric tourists appears to be distinctive for Canadians travelling abroad for bariatric surgery, it requires additional unpacking. Perceptions of stigma and isolation from friends and family, as well as health care professionals, impact the information seeking that potential bariatric tourists can engage in. Further, these perceptions of stigma are likely to be experienced whether the individual seeks the care in Canada or abroad. But this experience of stigma becomes additionally problematic in bariatric tourism when it limits the information individuals can obtain. This may be particularly problematic in Canada, where family doctors act as gatekeepers to access to more specialized care (Chan & Austin, 2003; Health Council of Canada, 2010; Romanow, 2002). When surgery is obtained domestically, it can be assumed that the patient’s primary care provider approves of the procedure through the act of referral to a surgeon. However, as is apparent from this study, support cannot be assumed or implied in the context of medical tourism for bariatric surgery. Lack of physician support may be for several reasons including but not limited to: (1) a physician’s belief that the surgical solution may not be appropriate for the patient; (2) a physician’s uncertainty about the quality of and standard practice of care abroad and; (3) a physician’s inexperience with providing follow-up care for bariatric patients or difficulty referring them on to appropriate specialists (Collier, 2014; Crooks et al., 2013; Runnels et al., 2014; Snyder et al., 2013). When self-navigation of care overlaps with a lack of support from patients’ regular care providers in Canada, the potential for exposure to health and safety risks such as care discontinuity are amplified through isolation from the Canadian health care system.

The findings show that when isolation from the Canadian health care system is coupled with the self-directed navigation of health care in Mexico, both the quantity and quality of advice and information these patients are receiving can be negatively impacted in the context of bariatric tourism. When individuals are faced with an unsupportive health care practitioner, it appears they are not able to confirm the accuracy of anecdotal information seeking with their regular health care practitioner. This becomes concerning as a central role of Canadian family doctors is to engage widely in patient education (Williams, Davis, Parker, & Weiss, 2002). This potential over-reliance on anecdotal or experiential evidence from previous patients may impart a false sense of security in prospective patients and limit the quantity of strong peer-reviewed evidence that patients would typically have access to in the domestic context. As a result, the risk that patients may not be giving truly informed consent is increased.

The final overlap of challenges that appears to be especially problematic occurs when challenges with follow-up care happen in conjunction with self-navigation of care. As shown in Figure 1, this intersection of challenges has been identified in previous medical tourism literature (e.g., Eissler & Casken, 2013; Johnston et al., 2013; Snyder et al., 2013), but is also present in a significant way in cases of bariatric tourism by Canadians. Research has shown that not having access to the extensive, life-long follow-up care that should be provided following bariatric surgery can be risky and have negative health impacts for these patients (Gould, Beverstein, Reinhardt, & Garren, 2007; Wheeler, Prettyman, Lenhard, & Tran, 2008; Zeigler, Sirveaux, Brunaud, Reibel, & Quilliot, 2009). The Canadian system for bariatric surgery has a significant pre and post-surgery programme with a specialized team of health care professionals, to ensure that patients are physically and psychologically ready for success with the surgery (Aarts et al., 2017; Karmali et al., 2010). This type of continuing care is much more challenging, if not impossible, in the context of medical tourism because of the spatial and temporal constraints of the transnational nature of care delivery which prevent patients from accessing follow-up care and therapy from the centre where they obtained surgery. Canadians who opt for bariatric surgery abroad must thus be proactive in assembling a follow-up team that can address their aftercare needs. Failure to do so means that health and safety risks of medical complications or significant disruptions to continuity of care are more likely.

### Future research directions

As was apparent from the interviews with former Canadian bariatric tourists, unless the qualification requirements change and surgery becomes otherwise more available throughout Canada (there is only evidence of increased surgical capacity in Ontario at this time), this practice of going abroad for care is likely to continue. Canadian bariatric tourists will continue to be exposed to health and safety risks to obtain the care they feel is necessary for their health. To be clear, we are not advocating to open this surgery to all that desire it. Rather, that given the growing obesity crisis globally, and in Canada, it might be time to reassess the qualification requirements for publicly funded surgery in Canada. This may mean changing the requirements or moving towards a case-by-case assessment of the overall impact on a person’s health the surgery would impart. Furthermore, given the significant amount of time participants spent discussing family doctors, including whether these physicians supported their decisions to go abroad for surgery, we believe an important direction for future research is to investigate the potential for informational interventions to be developed to inform this provider group about trends and key issues associated with bariatric tourism. While primary care providers are not directly providing follow-up care, increased awareness of the challenges associated with accessing bariatric surgery abroad may help to reduce the negative feelings prospective patients experience from their primary care physicians. In this way Canadian family doctors may be better situated to help their patients through the decision-making process even if they continue to engage in bariatric tourism. Furthermore, better equipping Canadian family doctors to guide patients through navigating this care could help to better educate patients on the reasons why these restrictions are in place in the first place. Family physicians will be enabled to better assess a patient’s readiness and/or motivation for the surgery, ability to independently lose weight beyond the surgery, and to engage in long term behavioural change, while also helping the patient to self-identify these aspects for themselves.

### Limitations

The study sought to recruit a particularly small and hard to reach population. Consequently, snowball sampling was heavily relied upon. This may limit the findings of this study as snowball sampling can result in participants sharing similar experiences due to similar sharing contexts (Magnani, Sabin, Saidel, & Heckathorn, 2005; Sadler, Lee, Lim, & Fullerton, 2010). While our recruitment did capture important aspects of difference among participants, such as in socio-economic status, there was no variation in the destination country for bariatric tourism. This works to limit the generalizability of our findings to other contexts as we captured no diversity in terms of destination location and experience. We also failed to recruit any participants from Canada’s most populous provinces, including Ontario. Recent changes to increase surgical capacity in Ontario may mean that more individuals requiring surgery are being approved for the procedures domestically and therefore fewer are travelling for care (CIHR, 2014; Christou, 2011; Christou & Efthimiou, 2009). However, this does not mean individuals from this province are not engaging in bariatric tourism.

## Conclusion

This paper has presented the findings of a thematic analysis derived from qualitative semi-structured interviews conducted with 20 Canadians who had previously privately obtained bariatric care outside of Canada. This paper sought to examine the challenges experienced by Canadian bariatric tourists that work in conjunction to heighten the health and safety risks patients experience beyond what they would normally experience should they have received the procedures domestically. Overall, it appears that these patients face a “triple threat” of challenges compared to if the surgery was performed domestically. This “triple threat” includes: (1) perceptions of stigma and isolation from family, friends, and the Canadian health care system which is coupled with (2) self-navigation of both the Canadian and Mexican health care system for information seeking and obtainment of care, and finally (3) a significant need for extensive life-long follow-up care which may or may not be achievable once the patient returns home. It appears that the conjunction of these challenges heightens the risk potential these patients experience. In the future, more research and examination into bariatric tourism by Canadian medical tourists is needed.

## Data Availability

The datasets used and/or analysed during the current study are available from the corresponding author on reasonable request.

## Note

1. The WHO classifies the health conditions of overweight and obesity based on body mass index (BMI). BMI is calculated as an individual’s weight (in kilograms) divided by their height squared (in metres squared). Overweight is defined as a BMI of 25–29.5 kg/m^2^. Obesity is defined as having a BMI greater than 30 kg/m^2^ (Buchwald et al., 2011; World Health Organization, 2018).
